# Longitudinal tract-based spatial statistics analysis of white matter diffusivity changes and cognitive decline during the transition from MCI to Alzheimer’s disease

**DOI:** 10.1371/journal.pone.0329893

**Published:** 2025-08-05

**Authors:** Sewon Lim, Hajin Kim, Youngjin Lee, for the Alzheimer’s Disease Neuroimaging Initiative

**Affiliations:** 1 Department of Health Science, General Graduate School of Gachon University, Yeonsu-gu, Incheon, Republic of Korea; 2 Department of Radiological Science, Gachon University, Yeonsu-gu, Incheon, Republic of Korea; Isfahan University of Medical Sciences, IRAN, ISLAMIC REPUBLIC OF

## Abstract

Longitudinal studies that analyze the changes in the axial diffusivity (AxD) and radial diffusivity (RD) values over time can elucidate the progression of white matter damage and its causal relationship with cognitive decline. This study aimed to investigate the longitudinal changes in white matter integrity based on AxD and RD and their association with cognitive decline in patients with mild cognitive impairment (MCI) that progressed to Alzheimer’s disease (AD). Eighteen participants diagnosed with MCI at baseline and AD at the follow-up examination were selected from the AD Neuroimaging Initiative and included in this 2-year study Tract-based spatial statistics (TBSS) was used to assess longitudinal changes in WM. Voxel-wise and region-of-interest (ROI) analyses were conducted, and statistical models controlled for age, sex, education, and APOE ε4 status. Correlation and multiple regression analyses were performed to examine the association between AxD/RD changes and changes in clinical dementia rating (CDR) scores. Significant increases in AxD and RD were observed over 2 years in widespread WM tracts, including the corpus callosum, internal capsule, corona radiata, cingulum, superior longitudinal fasciculus, and fornix. AxD changes, particularly in the left retrolenticular internal capsule, left posterior corona radiata, left fornix, and right superior longitudinal fasciculus, showed significant correlations with cognitive decline. In contrast, RD changes were not significantly associated with CDR changes in any region. Multivariate regression analysis identified AxD in the left retrolenticular internal capsule as a significant independent predictor of CDR changes. AxD was sensitive to microstructural alterations in WM associated with cognitive decline during the transition from MCI to AD and may serve as a valuable biomarker for early detection and monitoring of AD progression. Longitudinal DTI analyses provide critical insights into the temporal dynamics of WM degeneration and its role in clinical deterioration.

## Introduction

Alzheimer’s disease (AD) is characterized by the accumulation of amyloid plaques and tau-related neurofibrillary tangles, leading to synaptic loss and brain atrophy, particularly in the hippocampus and temporal lobes [1–5]. Additionally, white matter (WM) degeneration can occur in AD. Elevated tau levels in the cerebrospinal fluid have also been linked to WM damage [6]. Disruptions in WM integrity, including demyelination, axonal loss, and a deficiency in plasmalogen, are critical contributors to cognitive impairment in AD [7–9]. Furthermore, previous magnetic resonance imaging (MRI) studies have highlighted substantial microstructural damage in the WM [10–12]. However, whether WM damage is an early pathological hallmark of AD remains unclear [13,14].

Structural MRI is used to visualize brain atrophy in patients with AD; however, it exhibits limitations in detecting subtle damage to the WM, particularly damage in the early stages of AD, when atrophy is less pronounced [15,16]. Notably, diffusion tensor imaging (DTI) can detect microstructural changes and the integrity of WM tracts in specific brain regions [17–20]. The diffusion of water typically aligns with the orientation of the fiber tracts, but damage to these tracts reduces this anisotropy [21]. Axial diffusivity (AxD) and radial diffusivity (RD) are DTI metrics [22]. AxD represents the rate of water diffusion along the longitudinal axis, corresponding to the principal eigenvector (λ_1_). RD reflects the rate of water diffusion along the perpendicular axes, representing the mean diffusion coefficients along the two axes orthogonal to λ_1_ [23]. AxD can detect axonal degeneration, whereas RD can detect changes related to demyelination. Therefore, diffusivity metrics play a crucial role in detecting AD [24–26]. AD is characterized by changes in the WM, resulting in the accumulation of amyloid beta, axonal damage, and Wallerian degeneration [27]. Variations in AxD can be used to detect early pathological changes, such as axonal damage and inflammatory responses [28,29]. Therefore, AxD and RD can be used to comprehensively evaluate the distinct pathological mechanisms in the WM.

Pathological changes emerging in the early stages and progressively worsening over time have been observed in patients with mild cognitive impairment (MCI) that progresses to AD [30]. This gradual progression underscores the importance of early detection and timely intervention, particularly in terms of elucidating the association between subtle structural changes in the brain and cognitive decline over time. However, the cross-sectional design of most previous studies limited their ability to capture the dynamic nature of the changes in WM over time. Longitudinal studies that analyze the changes in AxD and RD over time can elucidate the progression of WM damage and its causal relationship with cognitive decline [31]. Furthermore, these analyses can provide insights into the pathological features of AD and evaluate whether WM damage can be used as an early marker of AD.

The present study hypothesized that 1) WM damage, as indicated by increased AxD and RD values, represents early pathological changes such as axonal degeneration and demyelination during the progression from MCI to AD, and 2) WM damage, as assessed by changes in AxD and RD, is associated with cognitive decline. The longitudinal changes in AxD and RD in patients with MCI that progressed to AD were evaluated using tract-based spatial statistics (TBSS) implemented using the FMRIB Software Library (FSL) over a 2-year period. Furthermore, the relationship between the assessed change trends and cognitive decline was analyzed.

## Materials and methods

### Study participants

**Fig 1:**
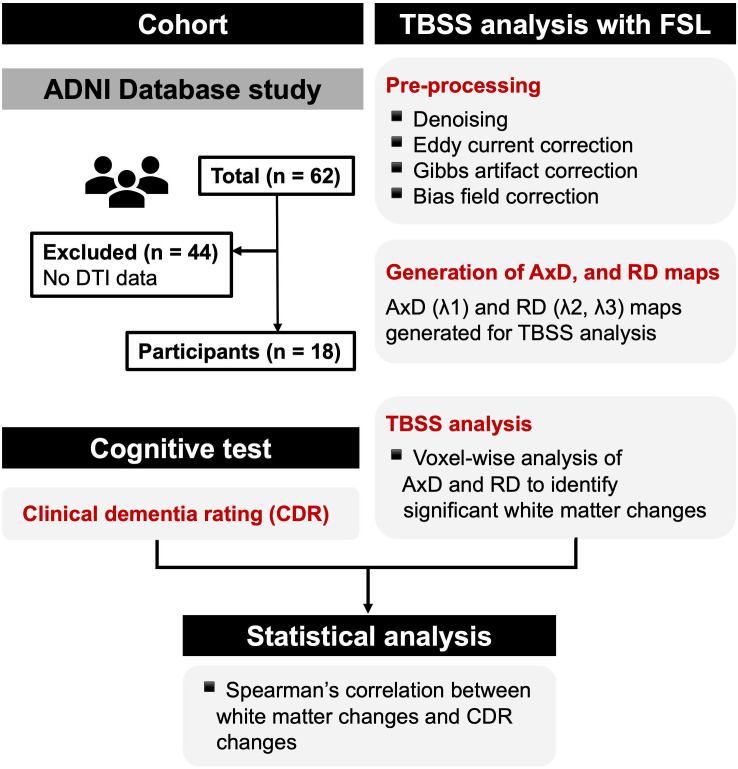
Flow diagram of the overall study: participant selection, TBSS analysis, and statistical analysis. DTI, diffusion tensor imaging; TBSS, tract-based spatial statistics; FSL, FMRIB Software Library; AxD, axial diffusivity; RD, radial diffusivity; CDR, clinical dementia rating.

Data sourced from the Alzheimer’s Disease Neuroimaging Initiative (ADNI) database (adni.loni.usc.edu) on 14 February 2024 were used in this study. The authors had no access to participant-identifying information during data collection or after study completion. Fig 1 provides an overview of the study design. Participants from the ADNI who had undergone DTI, cognitive assessments, and demographic evaluations at baseline and the 2-year follow-up examination were included in this study. Follow-up assessments were conducted at 24.78 ± 1.75 months after the baseline assessment. Only the participants diagnosed with MCI at baseline that progressed to AD at the follow-up examination were selected (n = 62). The participants with missing baseline or follow-up DTI images were excluded (n = 44). To evaluate the potential for sample bias, we compared the demographic and clinical characteristics of the included and excluded participants. Finally, 18 participants (age, 72.00 ± 8.69; 6 [33.33%] females) were included in the final analysis. Participants with ≥1 apolipoprotein E (APOE) ε4 allele were classified as carriers, whereas those with no APOE ε4 alleles were classified as non-carriers. The requirement for written informed consent was waived by Institutional Review Board of Gachon University (1044396–202405-HR-086–01).

### Neuropsychological assessment

The severity of dementia was assessed based on the clinical dementia rating (CDR). The CDR combines data from patient and caregiver interviews across six cognitive domains to assess cognitive impairment. The participants were classified into cognitively normal, MCI, and AD groups at both time points based on the CDR scores, in accordance with the ADNI criteria (https://adni.loni.usc.edu/methods/documents/). Cognitive decline was defined as the change in the CDR scores over the 2-year period (cognitive decline = CDR at follow-up − CDR at baseline).

### Acquisition of diffusion tensor imaging and processing

A 3T MRI scanner was used to acquire the DTI data for each participant. Detailed imaging protocols have been described in publicly available documents (https://adni. loni. usc. edu/methods/documents/mri-protocols). FSL version 6.0.6 (http://www.fmrib.ox.ac.uk/fsl) and MRtrix3 (https://www.mrtrix.org) were used to correct DTI artifacts, such as noise, Gibbs artifacts, eddy current-induced distortions, and bias field inhomogeneity [32]. Subsequently, the diffusion tensor model was fitted using “dtifit” in the FSL to generate the λ_1_ (L1), λ_2_ (L2), and λ_3_ (L3) maps. The primary eigenvalue (λ_1_) was used to determine the AxD, whereas the secondary eigenvalues (λ_2_ and λ_3_) were averaged to derive the RD.

### TBSS

TBSS is an automated analysis method used to evaluate the changes in fractional anisotropy (FA) in the cerebral WM. It employs voxel-wise statistics on diffusion indices while utilizing conventional voxel-based analysis techniques [33]. TBSS was selected for its robustness in aligning white matter tracts across participants and its suitability for small longitudinal datasets. It enables voxel-wise statistical analysis while minimizing the impact of spatial misregistration. However, one limitation of TBSS is that it restricts the analysis to the central white matter skeleton, potentially overlooking peripheral or crossing fiber regions where early pathological changes may also occur [34]. Future studies may benefit from complementary approaches such as fixel-based analysis or tractometry to capture a broader range of white matter alterations. Voxel-wise analysis of FA data derived from DTI images was conducted using TBSS as a preliminary step for analyzing AxD and RD. Although FA is a widely used metric in TBSS and was used in this study to construct the white matter skeleton, we did not include FA in the correlation or regression analyses. This is because FA reflects a composite of axial and radial diffusivity and may lack specificity in isolating pathological mechanisms [35]. Therefore, we focused our analyses on AxD and RD to better elucidate the distinct microstructural changes occurring in AD. The FSL was used to register the FA maps to an FA template space (FMRIB58_FA 1 × 1 × 1 mm), created using the FA maps of the 18 participants in this study. A mean FA skeleton representing the core of the WM tracts shared among the participants was constructed by averaging the FA maps. A threshold of 0.2 was applied to refine the skeleton such that only voxels with sufficient FA values, indicating WM integrity, were included in the analysis. The normalized FA data of each participant were mapped onto this skeleton to facilitate the voxel-wise statistical analysis of cross-subject differences using general linear modeling [36]. The same skeleton and registration parameters were applied to the AxD and RD maps to ensure that these metrics were evaluated within the same structural framework. The Johns Hopkins University white-matter tractography atlas was used to conduct the region-of-interest (ROI) analysis. ROI masks were applied to the corresponding key WM tracts, including the cingulum (CG), corpus callosum (CC), and uncinate fasciculus, to extract the AxD and RD values [37]. ROI analyses were conducted in an exploratory manner based on regions that showed significant differences in AxD and RD in the TBSS whole-brain analysis. Therefore, the ROI-level statistical tests should be interpreted with caution, as they were derived post hoc rather than based on a priori hypotheses. This approach facilitated a detailed examination of the microstructural changes within anatomically defined regions that complemented the voxel-wise statistical analysis [38].

### Statistical analysis

All statistical analyses were conducted using Jamovi (version 2.3.28.0; retrieved from https://www.jamovi.org) and FSL (version 6.0.6; https://www.fmrib.ox.ac.uk/fsl). Longitudinal differences in the AxD and RD values across the WM tracts were analyzed using TBSS with permutation-based statistical testing.

TBSS is a voxel-wise statistical technique to improve WM skeleton alignment by applying nonparametric permutation testing to evaluate group variations and avoid arbitrary spatial smoothing. In this study, permutation testing with 5,000 iterations generated a null distribution of t-values by randomly rearranging the data among the participants [39]. Statistical significance was ascertained by matching the observed t-values to the null distribution and comparing them to a preset threshold. A threshold-free cluster enhancement method was applied within the permutation-based paired t-tests to correct for multiple comparisons at the voxel level. Statistical significance was set at **P* *< 0.05 and evaluated using threshold-free cluster enhancement-corrected *P*-values [40]. To account for potential confounding factors, a general linear model was incorporated into the TBSS framework, with age, sex, education, and APOE ε4 status as independent variables and AxD/RD as the dependent variable [41–43].

Spearman’s correlation analysis was used to examine changes in ROI-based AxD, RD, and CDR in specific WM regions while controlling for age, sex, education, APOE ε4 status, and baseline CDR score. Given the small sample size, applying the Bonferroni correction to the correlation analyses would have significantly lowered the statistical power, eliminating significant associations. Therefore, uncorrected *P*-values were reported to investigate possible patterns in the connection between clinical progression and changes in WM integrity. To further evaluate the adequacy of the sample size in detecting meaningful correlations, a post hoc power analysis was conducted using G*Power (version 3.1).

Furthermore, independent contributions of the AxD and RD changes to clinical development were evaluated using multiple linear regression analysis. The change in CDR scores was the dependent variable for this analysis, whereas AxD and RD were the independent variables. Age, sex, education, APOE є4 status, and baseline CDR score were treated as confounders in the regression model. Multivariate regression analysis was conducted independently for each WM region that showed notable variations in AxD or RD in the TBSS analysis.

## Results

### General demographics

Table 1 presents the demographic data. A statistically significant increase (*P* < 0.001) was observed in the CDR score over 2 years, from a mean score of 2.42 ± 1.17 at baseline to 4.64 ± 1.44 at the follow-up, indicating substantial cognitive decline. To assess potential sample bias due to exclusion of participants with missing DTI data, demographic and clinical characteristics were compared between the included (n = 18) and excluded (n = 44) groups. No significant differences were found in age (P = 0.909), sex (P = 0.578), education level (P = 0.967), APOE ε4 carrier status (P = 0.251), and baseline CDR (P = 0.074).

**Table 1:** Demographic characteristics of the participants and the cognitive assessment score.

	Baseline	Follow-up	*P*
Age, mean (SD), y	72.00 (8.69)	74.00 (8.69)	<0.001
Sex			
Female, n (%)	6 (33.33)	–	–
Male, n (%)	12 (66.67)	–	–
Education (y)			
≤9, n (%)	0 (0.00)	–	–
10–12, n (%)	2 (11.11)	–	–
13–16, n (%)	7 (38.89)	–	–
>16, n (%)	9 (50.00)	–	–
APOE є4 carrier status			
Carrier, n (%)	6 (33.33)	–	–
Non-carrier, n (%)	12 (66.67)	–	–
CDR	2.42 ± 1.17	4.64 ± 1.44	<0.001

Participants with APOE ε4 carrier status ≥1 were classified as carriers, whereas those with no APOE ε4 alleles were classified as non-carriers. Variables that were constant over time (e.g., sex, education, APOE ε4 status) are reported at baseline only. P-values represent comparisons between baseline and follow-up for longitudinal variables. SD, standard deviation; APOE, apolipoprotein; CDR, clinical dementia rating.

### Differences in WM integrity

**Fig 2:**
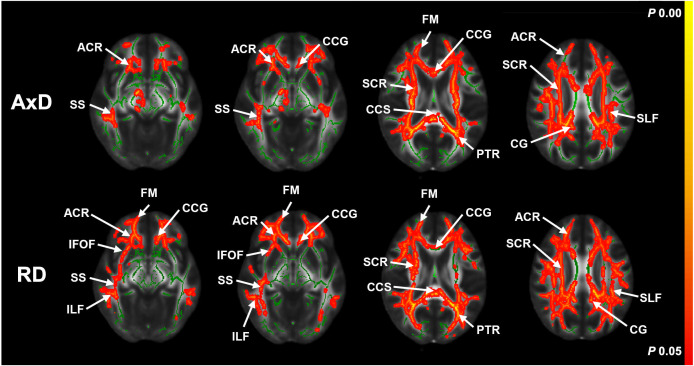
TBSS results showing significant AxD and RD changes at baseline and the follow-up. The regions indicated in green represent the mean white matter skeleton, whereas the regions indicated in red represent the regions with increased AxD (first row) and RD (second row). The decreased diffusivity metrics (AxD and RD) exhibited no statistically significant differences at *P* < 0.05 after correcting for multiple comparisons (FWE). The color intensity follows the Red–Yellow scale, where red represents stronger statistical significance (closer to *P* = 0.05), and the color transitions towards yellow as the *P*-value approaches zero. TBSS, tract-based spatial statistics; AxD, axial diffusivity; RD, radial diffusivity; FWE, family-wise error; FM, forceps minor; ACR, anterior corona radiata; CCS, splenium of corpus callosum; PCR, posterior corona radiata; SS, sagittal stratum; CCG, genu of corpus callosum; SCR, superior corona radiata; PTR, posterior thalamus radiation; CG, cingulum cingulate gyrus; SLF, superior longitudinal fasciculus; IFOF, inferior fronto-occipital fasciculus; ILF, inferior longitudinal fasciculus.

Compared with those observed at baseline, TBSS analysis revealed a significant increase in the AxD and RD values at the follow-up examination across extensive regions of the brain, such as the CC, internal capsule (IC), corona radiata (CR), thalamic radiation (TR), lateral temporoparietal regions, longitudinal fasciculus, sagittal and external capsule, CG, fornix, and tapetum (Fig 2).

Table 2 summarizes the specific WM regions exhibiting significant changes in the AxD and RD values, as well as the number of voxels affected. Specifically, the AxD and RD values of the CC exhibited extensive changes. The largest voxel sizes for the increase in AxD were observed in the superior longitudinal fasciculus (SLF) and posterior CR (PCR). Widespread increases were observed in other areas such as the IC, CR, and CG, indicating considerable microstructural changes during the two-year follow-up period.

**Table 2:** Anatomic locations exhibiting significant changes in AxD and RD.

	Voxel size	
White matter region	AxD	RD
Genu of corpus callosum	736	867
Body of corpus callosum	1073	805
Splenium of corpus callosum	1625	1387
Cerebral peduncle	78	
Anterior limb of internal capsule R	282	154
Anterior limb of internal capsule L	353	
Posterior limb of internal capsule R	355	36
Posterior limb of internal capsule L	155	
Retrolenticular part of internal capsule R	375	261
Retrolenticular part of internal capsule L	400	69
Anterior corona radiata R	1090	1227
Anterior corona radiata L	964	1164
Superior corona radiata R	1196	1264
Superior corona radiata L	1124	784
Posterior corona radiata R	628	598
Posterior corona radiata L	623	581
Posterior thalamic radiation R	545	641
Posterior thalamic radiation L	406	536
Sagittal stratum R	204	235
External capsule R	341	419
External capsule L	368	
Cingulum cingulate gyrus R	76	122
Cingulum cingulate gyrus L	19	134
Fornix cres stria terminalis R	10	
Fornix cres stria terminalis L	38	
Superior longitudinal fasciculus R	1025	1171
Superior longitudinal fasciculus L	455	791
Inferior fronto occipital fasciculus R	21	206
Inferior fronto occipital fasciculus L	16	37
Uncinate fasciculus R	21	
Uncinate fasciculus L	21	
Tapetum R	25	28
Tapetum L	13	16

AxD, axial diffusivity; RD, radial diffusivity; FWE, family-wise error; R, right; L, left.

### Correlation between WM integrity and cognitive decline

**Fig 3:**
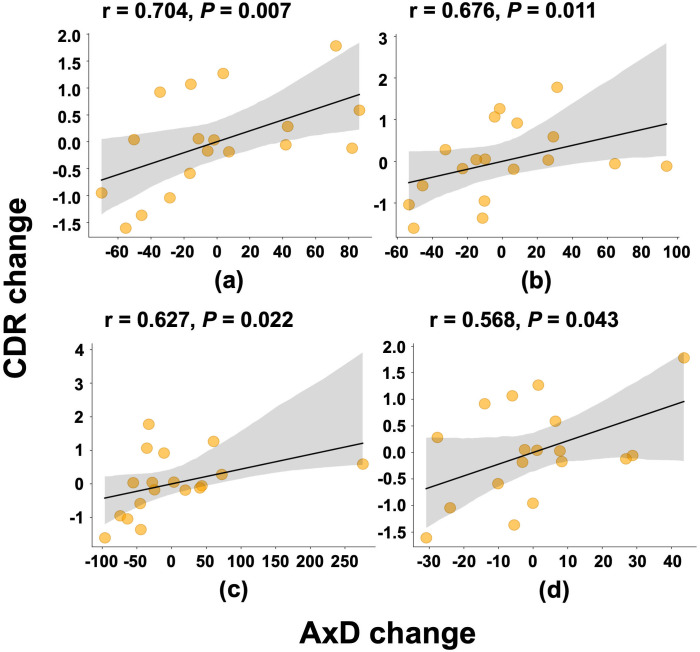
Partial correlation plots showing the association between AxD change and CDR change in four significant white matter regions: (a) left RIC, (b) left PCR, (c) left FST, and (d) right SLF. All values were residualized after adjusting for age, sex, education, APOE ε4 status, and baseline CDR. Solid black lines indicate the linear trend, and shaded areas represent the 95% confidence intervals. Each dot corresponds to an individual participant. Spearman’s correlation coefficients and P values are reported above each panel. AxD, axial diffusivity; RIC, retrolenticular part of internal capsule; PCR, posterior corona radiata; FST, fornix cres stria terminalis; SLF, superior longitudinal fasciculus.

Table 3 presents the significant associations in specific regions observed in the correlation analysis between WM integrity and cognitive decline. A detailed summary of individual patient-level AxD and RD changes is provided in S1 Table. AxD changes exhibited the strongest correlation with the changes in CDR score. Significant correlations were observed in regions such as the left retrolenticular part of the IC (*r* = 0.704, **P* *= 0.007), left PCR (*r* = 0.676, *P* = 0.011), left fornix cres stria terminalis (**r* *= 0.627, *P* = 0.022), and right SLF (*r* = 0.568, *P* = 0.043). These relationships are further illustrated in Fig 3, which displays scatter plots of residualized AxD and CDR change values after adjusting for age, sex, education, APOE4 status, and baseline CDR. Post hoc power analysis using G*Power (version 3.1) indicated that the study had 93.7% power to detect a correlation of r = 0.704, and 86.3% power for the average observed correlation across all significant regions (mean r = 0.644), with α = 0.05 and N = 18. No significant correlations were observed between the changes in RD and changes in CDR scores across any of the examined WM regions.

**Table 3:** Correlation between AxD and RD in specific white matter regions and CDR scores.

	AxD		RD	
White matter region	*r*	*P*	*r*	*P*
Genu of corpus callosum	−0.267	0.378	−0.349	0.243
Body of corpus callosum	0.217	0.476	0.021	0.947
Splenium of corpus callosum	0.171	0.577	−0.037	0.905
Cerebral peduncle	−0.225	0.460		
Anterior limb of internal capsule R	0.393	0.468	0.233	0.443
Anterior limb of internal capsule L	0.501	0.081		
Posterior limb of internal capsule R	−0.248	0.415	0.342	0.253
Posterior limb of internal capsule L	0.429	0.144		
Retrolenticular part of internal capsule R	0.152	0.621	−0.005	0.986
Retrolenticular part of internal capsule L	0.704	0.007^*^	0.196	0.522
Anterior corona radiata R	−0.298	0.323	−0.041	0.895
Anterior corona radiata L	0.123	0.690	−0.247	0.416
Superior corona radiata R	0.264	0.383	0.082	0.789
Superior corona radiata L	0.351	0.240	0.329	0.272
Posterior corona radiata R	0.337	0.260	−0.067	0.828
Posterior corona radiata L	0.676	0.011^*^	0.479	0.098
Posterior thalamic radiation R	0.197	0.519	−0.101	0.743
Posterior thalamic radiation L	0.453	0.120	0.482	0.095
Sagittal stratum R	0.289	0.338	0.132	0.667
External capsule R	0.264	0.384	0.013	0.966
External capsule L	0.117	0.704		
Cingulum cingulate gyrus R	0.128	0.677	0.336	0.262
Cingulum cingulate gyrus L	0.093	0.763	0.144	0.638
Fornix cres stria terminalis R	0.407	0.167		
Fornix cres stria terminalis L	0.627	0.022^*^		
Superior longitudinal fasciculus R	0.568	0.043^*^	0.193	0.527
Superior longitudinal fasciculus L	0.521	0.068	0.396	0.180
Inferior fronto occipital fasciculus R	−0.106	0.731	−0.375	0.207
Inferior fronto occipital fasciculus L	−0.258	0.395	−0.364	0.221
Uncinate fasciculus R	−0.123	0.689		
Uncinate fasciculus L	−0.301	0.318		
Tapetum R	−0.071	0.817	0.077	0.801
Tapetum L	−0.097	0.752	0.083	0.322

AxD, axial diffusivity; RD, radial diffusivity; CDR, clinical dementia rating; R, right; L, left.

### Multivariate regression analysis of WM integrity and changes in CDR scores

We performed multiple linear regression analyses with CDR changes as the dependent variable, adjusting for age, APOE є4 status, education, sex, and baseline CDR, to examine the independent contributions of changes in AxD and RD to clinical progression. The regression analysis involves ROIs exhibiting significant differences in both AxD and RD values in the TBSS analysis. Notably, AxD changes in the left retrolenticular IC were significantly associated with CDR changes (β [95% confidence interval (CI)] = 0.0141 [0.0005, 0.0277], *P* = 0.044). Additionally, AxD changes in the right SLF (β [95% CI] = 0.0230 [−0.0069, 0.0528], *P* = 0.117), right sagittal stratum (β [95% CI] = 0.0157 [−0.0068, 0.0382], *P* = 0.152), left SLF (β [95% CI] = 0.0144 [−0.0074, 0.0361], **P* *= 0.172), and left posterior TR (β [95% CI] = 0.0093 [−0.0056, 0.0242], *P* = 0.194) exhibited weak positive trends with CDR changes, although these associations were not statistically significant. S2 Table summarizes the results of regression analysis for all ROIs.

## Discussion

Anisotropic diffusion metrics measured using DTI, such as AxD and RD, are sensitive to microstructural barriers and obstacles (e.g., cell membranes, myelin sheaths, and microtubules) [44]. These metrics are widely recognized as indicators of WM integrity, reflecting WM damage [45]. Therefore, the changes in the WM microstructure were longitudinally evaluated by analyzing the diffusion metrics—AxD and RD. Furthermore, their associations with cognitive decline were also assessed. TBSS analysis revealed a significant increase in AxD and RD in several WM regions, such as the CC, CR, and SLF. The increase in AxD was widespread, and it correlated with cognitive decline. Conversely, RD changes across all examined WM regions did not significantly associate with CDR changes. These findings indicated that an increase in AxD is closely related to cognitive decline.

TBSS analysis revealed significant changes in AxD and RD across the CC, CR, CG, SLF, inferior fronto-occipital fasciculus (IFOF), and IC. The CC, the main WM tract connecting the left and right hemispheres of the brain, plays a critical role in maintaining cognitive function [46]. It enables efficient transmission and coordination of information between the two hemispheres, thereby facilitating the development and maintenance of cognitive abilities [47]. Particularly, the CC enables smooth communication between the hemispheres by transmitting electrical and chemical signals, thereby playing an essential role in attention, memory, language processing, spatial perception, and emotional regulation [48]. Consequently, damage to the CC can weaken the connection between the hemispheres, thereby impairing memory, attention, language processing, and other cognitive functions [49]. The CR, a part of the limbic-thalamocortical circuitry, is a vital pathway that connects the cerebral cortex with the subcortical structures. The CR facilitates communication and information exchange essential for cognitive functions, such as memory and language processing [48]. It supports sensory, motor, and cognitive control. Changes in its integrity, particularly in the ACR, can impair prefrontal cortex functions involved in cognitive control [50]. Damage to the CG, a critical component of the limbic system, is often observed in patients with AD, along with an increase in RD in the CG, indicating disruption of WM integrity [51]. These changes may disrupt connections between brain regions within the limbic system, affecting emotion regulation, memory, and cognitive processes [51,52]. The SLF, which connects the prefrontal, temporal, and occipital cortices through the posterior parietal cortex, features extensive bidirectional pathways [53]. It plays a critical role in maintaining motor coordination and gait function [54]. Therefore, damage to the SLF can impair the motor coordination system, leading to difficulties in walking and balance [55]. The IFOF links the frontal, temporal, and occipital lobes, thereby enabling the integration of auditory and visual information and contributing to higher-order cognitive processing [56]. Disruption of the IFOF can impair multisensory integration and complex cognitive functions [57]. The IC, a bilateral WM structure located in the inferomedial region of each hemisphere, carries afferent and efferent fibers to and from the cerebral cortex [58]. Notably, the posterior limb of the IC contains fiber pathways involved in language and cognitive functions [48]. Therefore, changes in the IC can result in extensive impairments in motor and sensory functions, in addition to deficits in language execution and cognitive processing [59]. These findings highlight the vulnerability of WM tracts to AD and suggest that damage to these tracts may lead to cognitive and functional impairments.

Previous cross-sectional studies have reported an increase in AxD and RD in the CC, SLF, and CG in patients with AD [60]. A longitudinal analysis of the progression from MCI to AD further demonstrated significant increases in AxD and RD in these regions, highlighting structural changes in the WM during disease progression. Diffusivity changes have also been observed in limbic system regions closely associated with lower cognitive scores, including the posterior CG, fornix, and CCS [61–64]. Notably, previous studies have reported significant correlations between cognitive scores, such as the Mini-Mental State Examination, and diffusivity changes in the posterior CG [65,66].

In the present study, AxD changes in the left retrolenticular region of the IC, left PCR, left fornix cres stria terminalis, and right SLF exhibited significant correlations with changes in CDR scores, indicating that axonal degeneration in these regions may contribute to cognitive decline. The involvement of the left retrolenticular region of the IC and left PCR, which are closely connected to the temporal lobe and posterior temporoparietal circuitry, is consistent with previous research highlighting the role of these pathways in cognitive processing and memory function [67–69]. The fornix, a major component of the limbic system, has also been widely associated with memory impairment and AD-related neurodegeneration [70]. Moreover, the SLF plays a crucial role in long-range cortical communication and has been implicated in executive function and visuospatial deficits in AD [71].

Interestingly, no significant correlations were observed between RD changes and CDR changes across any of the examined WM regions in the present study. One possible explanation is that axonal degeneration, rather than myelin integrity loss, plays a predominant role in clinical progression at this stage of the disease. Increases in AxD may primarily reflect severe tissue loss, widespread structural damage, and increased extracellular space, rather than direct demyelination [72]. The observed AxD increases may reflect Wallerian degeneration, leading to progressive axonal atrophy [73,74]. Axonal loss and inflammatory responses, particularly microglial activation, have been proposed as key mechanisms underlying the pathological progression of AD, potentially explaining the strong association between AxD changes and cognitive decline in the present study [26,74]. Furthermore, AxD changes emerge earlier than RD changes during AD development [26,75]. In contrast, RD primarily reflects myelin degeneration, which may become more apparent in the later stages of AD progression. The lack of significant RD–CDR associations in this study may therefore be attributed to the temporal dynamics of pathology, with demyelination following earlier axonal damage. Additionally, technical limitations such as the spatial resolution of DTI and the skeleton-based nature of TBSS may hinder the detection of subtle peripheral WM changes where myelin loss typically occurs. Previous studies have reported mixed findings regarding RD changes in AD, with some suggesting that RD alterations are less pronounced than AxD in early stages [76], while others reported associations primarily in advanced disease [45]. These considerations underscore the importance of multimodal and longitudinal approaches to better disentangle the distinct pathological signatures captured by different DTI metrics. Therefore, AxD could be a sensitive indicator of early pathological changes. In this study, the changes in AxD exhibited a significant correlation with the changes in CDR in regions, including the left retrolenticular region of the IC, left PCR, left fornix cres stria terminalis, and right SLF.

Multivariate regression analysis was used to investigate the separate effects of AxD and RD changes on clinical progression. AxD changes in the left retrolenticular IC correlated with CDR changes, implying that axonal degeneration in this area plays a role in cognitive decline. Although these correlations did not exhibit statistical significance, AxD increases in the right SLF, right SS, left SLF, and left PT showed minor positive trends with CDR changes.

Our findings suggest that AxD changes, particularly in the left retrolenticular IC, serve as independent predictors of cognitive decline, reinforcing the hypothesis that axonal damage is the primary driver of microstructural alterations in AD progression. The significant association between AxD changes in this region and CDR reduction emphasizes the importance of axonal integrity in preserving cognitive function. These findings further imply that although axonal degeneration is clearly linked to cognitive decline, not all WM tracts equally contribute to disease progression, and regional specialization is essential in AD-related pathology. In contrast, the changes in RD across all examined WM regions were not substantially associated with CDR changes. RD is primarily associated with myelin damage; therefore, myelin loss in AD may contribute to cognitive decline by disrupting WM integrity, but it may not be the primary driver of disease progression. AxD reflects axonal pathology, and RD captures myelin-related abnormalities. Consequently, the correlation between AxD and RD observed in particular regions with cognitive decline suggests that both diffusion metrics are sensitive indicators of pathological progression [63]. While changes in AxD reflect early pathological progression, changes in RD become more prominent in the later stages of the disease progression. Therefore, AxD may be a reliable marker for identifying and monitoring the initial progression of AD, particularly given its independent association with cognitive decline observed in the present study [26,67,74].

The progression from MCI to AD was longitudinally analyzed in the present study, and the microstructural changes in WM integrity were evaluated to assess subtle pathological changes in the WM. In contrast to previous cross-sectional studies that revealed pathological changes at a specific time point, longitudinal studies, such as the present study, provide a clearer understanding of the temporal progression of these changes and their association with cognitive decline. Therefore, AxD, which offers important insights into the underlying pathology of AD, may be a valuable biomarker for early diagnosis and tracking of disease progression. Although AxD demonstrates potential as an early biomarker of axonal degeneration in AD, its clinical translation requires further work. Specifically, standardization across imaging protocols and scanners is necessary to ensure reproducibility. In addition, normative datasets should be established to define AxD thresholds indicative of pathological changes. Future studies should also assess the cost-effectiveness and clinical utility of incorporating AxD metrics into routine diagnostic workflows.

This study has some limitations. First, changes in AxD values have been inconsistently reported across studies, with both increases and decreases being observed. This inconsistency may be attributed to multiple pathological processes, including axonal damage, inflammatory responses, and changes in the extracellular space, affecting AxD. Therefore, further studies are warranted to clarify the extent to which AxD reflects specific pathological processes. Second, although AxD and RD reflect axonal damage and myelin degeneration, respectively, they rely on simplified assumptions of the diffusion tensor model. Therefore, the complexity of actual WM structures may not be captured completely. Advanced techniques, such as neurite orientation dispersion and density imaging or multi-shell DTI, must be used in future studies to overcome these limitations. Third, the TBSS analysis in this study only focused on the core regions of the WM tract, projecting the highest FA values onto the alignment-invariant tract representation (the FA skeleton) for statistical analysis. This approach improves the precision of the analysis; however, subtle changes occurring outside the skeleton may have been overlooked. Therefore, these findings may not fully represent all structural changes in the WM. Fourth, cognitive function was evaluated using CDR scores only. CDR is a useful tool for assessing the severity of AD; however, it lacks a detailed assessment of various cognitive domains. Additional neuropsychological tools, such as the Montreal Cognitive Assessment or Mini-Mental State Examination, must be incorporated in future studies to comprehensively assess multidimensional cognitive changes. Fifth, the small sample size (n = 18) limits the generalizability of the findings, as a small sample size reduces the statistical power and increases the likelihood of overlooking subtle changes. Therefore, future studies with larger sample sizes must be conducted to validate these findings and improve their reliability. Additionally, although uncorrected p-values were reported due to the small sample size, we acknowledge the risk of inflated false-positive rates. Therefore, future studies should validate our findings in larger samples using more stringent correction methods such as False Discovery Rate. Additionally, ROI analyses were conducted post hoc based on TBSS findings, which may raise concerns about circular reasoning. Although these exploratory analyses provided further insights into regional associations with cognitive decline, future studies should employ a priori ROI definitions or independent datasets for validation to enhance the robustness of the findings. Another limitation of this study is the exclusive use of the CDR as a cognitive measure. While CDR is a valid instrument for staging the severity of dementia, it does not capture domain-specific cognitive deficits. Future studies should incorporate neuropsychological batteries that assess memory, language, attention, and executive function to more precisely elucidate the relationship between white matter microstructural changes and cognitive decline. Lastly, although APOE ε4 status was included as a covariate in all statistical models, potential interaction effects between ε4 status and white matter changes were not tested in this study. Due to the limited sample size and small number of ε4 carriers, such analyses may have been underpowered. Further studies with larger samples should explore whether APOE ε4 status moderates the relationship between DTI metrics and cognitive decline.

A significant correlation between WM microstructural changes assessed using diffusion metrics, including AxD and RD, and cognitive decline was observed during the progression from MCI to AD in this longitudinal study. The findings of the present study demonstrate that an increase in AxD is particularly prevalent and strongly associated with changes in cognitive status. Therefore, AxD may be a sensitive indicator of early pathological changes. Furthermore, an increase in RD was observed in fewer regions. Collectively, the findings of the present study highlight the utility of AxD as a potential biomarker for the early detection and monitoring of AD progression, providing insights into the distinct role of WM diffusion metrics reflecting axonal and myelin pathology.

## Supporting information

S1 table AxD and RD changes in white matter ROIs for individual participants. (XLSX)

S2 table ROI-wise multivariate regression analysis of AxD and RD changes on CDR score changes. (XLSX)

S3 File Acknowledgment list of ADNI investigators. (PDF)
